# Investigation of sex differences in the expression of *RORA* and its transcriptional targets in the brain as a potential contributor to the sex bias in autism

**DOI:** 10.1186/2040-2392-6-7

**Published:** 2015-05-13

**Authors:** Valerie W Hu, Tewarit Sarachana, Rachel M Sherrard, Kristen M Kocher

**Affiliations:** Department of Biochemistry and Molecular Medicine, The George Washington University School of Medicine and Health Sciences, 2300 Eye St. NW, Washington, DC 20037 USA; Department of Clinical Chemistry, Faculty of Allied Health Sciences, Chulalongkorn University, Bangkok, Thailand; Institut de Biologie Paris Seine, Sorbonne Universités, UPMC Univ Paris 06 & CNRS, UMR 8256 Biological Adaptation and Ageing, F-75005 Paris, France

**Keywords:** Autism, *RORA* expression, Transcriptional targets, Sex differences, Postmortem brain tissues, Mouse brain

## Abstract

**Background:**

Autism spectrum disorder (ASD) is a neurodevelopmental condition characterized by significant impairment in reciprocal social interactions and communication coupled with stereotyped, repetitive behaviors and restricted interests. Although genomic and functional studies are beginning to reveal some of the genetic complexity and underlying pathobiology of ASD, the consistently reported male bias of ASD remains an enigma. We have recently proposed that retinoic acid-related orphan receptor alpha (*RORA*), which is reduced in the brain and lymphoblastoid cell lines of multiple cohorts of individuals with ASD and oppositely regulated by male and female hormones, might contribute to the sex bias in autism by differentially regulating target genes, including *CYP19A1* (aromatase), in a sex-dependent manner that can also lead to elevated testosterone levels, a proposed risk factor for autism.

**Methods:**

In this study, we examine sex differences in RORA and aromatase protein levels in cortical tissues of unaffected and affected males and females by re-analyzing pre-existing confocal immunofluorescence data from our laboratory. We further investigated the expression of *RORA* and its correlation with several of its validated transcriptional targets in the orbital frontal cortex and cerebellum as a function of development using RNAseq data from the BrainSpan Atlas of the Developing Human Brain. In a pilot study, we also analyzed the expression of *Rora* and the same transcriptional targets in the cortex and cerebellum of adult wild-type male and female C57BL/6 mice.

**Results:**

Our findings suggest that *Rora/RORA* and several of its transcriptional targets may exhibit sexually dimorphic expression in certain regions of the brain of both mice and humans. Interestingly, the correlation coefficients between *Rora* expression and that of its targets are much higher in the cortex of male mice relative to that of female mice. A strong positive correlation between the levels of RORA and aromatase proteins is also seen in the cortex of control human males and females as well as ASD males, but not ASD females.

**Conclusions:**

Based on these studies, we suggest that disruption of *Rora*/*RORA* expression may have a greater impact on males, since sex differences in the correlation of *RORA* and target gene expression indicate that RORA-deficient males may experience greater dysregulation of genes relevant to ASD in certain brain regions during development.

**Electronic supplementary material:**

The online version of this article (doi:10.1186/2040-2392-6-7) contains supplementary material, which is available to authorized users.

## Background

Autism spectrum disorder (ASD) refers to a group of neurodevelopmental disorders that are diagnosed on the basis of impaired social interactions and communication, especially social reciprocity, and the presence of aberrant, repetitive, and stereotyped behaviors [1]. Because of the strong heritability of ASD based on monozygotic twin and sibling studies [2, 3], there have been extensive searches for genetic mutations and variations that may cause ASD [4–13]. To date, there are hundreds to thousands of genes that are considered autism candidate or susceptibility genes in the autism gene databases [14, 15], with the number of genes continually growing. However, the combined genetic variations account for only approximately 20% of the cases, with no single gene or genetic variation associated with more than 1% of the cases. Thus, the etiology of the majority of ASD cases is still unknown.

Interestingly, ASD is consistently reported as having a higher incidence (approximately 4:1) in males than in females [16]. Yet, the mechanism for the sex bias is unknown. Several hypotheses for the sex bias in ASD include: (1) genetic mechanisms which might involve (a) genes on the X or Y chromosomes; (b) skewed X-inactivation, (c) sex-specific imprinting defects on either the X chromosome or autosomes; (2) the extreme male brain hypothesis which posits that elevated fetal testosterone is a risk factor for ASD; and (3) gene-environment interactions that predispose an individual to ASD. All of these hypotheses have been comprehensively described in several recent reviews [17–19]. To date, none of these hypotheses, discussed briefly in the next paragraph, have been either proven or disproven. Given the clinical and genetic heterogeneity of ASD, it is possible that each of these mechanisms for sex bias may apply to specific cohorts of individuals with ASD. What is clear, however, is that a validated mechanism for sex bias will reveal a fundamental process inherent to the core biology of ASD.

With regard to genetic mechanisms for sex bias, there are a few candidate genes for ASD on the sex chromosomes, but the reported genetic variations in them cannot account for the majority of cases. On the other hand, the extreme male brain hypothesis proposed by Baron-Cohen and colleagues focuses on elevated testosterone levels as a risk factor for ASD. This hypothesis has been investigated primarily by correlating autistic behaviors and traits with fetal testosterone levels in amniotic fluid during gestation of the individuals [17, 20–23]. More recently, this group has investigated differences in brain morphology linked to circulating testosterone levels in adults. Their studies demonstrated both morphological and volumetric changes in the brain that are both sex-dependent [24] as well as dependent on diagnosis of ASD [25, 26]. Direct investigation of the third hypothesis regarding gene by environment (GxE) interactions that may predispose an individual to ASD has been hampered by lack of knowledge regarding the genes that may be involved and the environmental factors that are relevant to ASD. We suggest that the reciprocal regulatory interaction between retinoic acid-related orphan receptor alpha (*RORA*) and sex hormones which includes RORA-mediated transcriptional regulation of *CYP19A1*, described below, may be a candidate for GxE interactions that modulate risk for ASD.

Our recent integrative genomic analyses of ASD involving gene expression and methylation profiling of lymphoblastoid cell lines (LCL) from monozygotic twins and pairs of siblings discordant for autism diagnosis revealed the dysregulated expression of many genes in ASD [27, 28], some of which have been associated with aberrant methylation of the gene promoters [29]. One of the downregulated and hypermethylated genes was the nuclear receptor *RORA*. Further analysis of this gene (which exhibits steroid hormone receptor binding sites in its promoter region) revealed that its expression was upregulated by estradiol but downregulated by the androgen dihydrotestosterone (DHT) [30]. Moreover, RORA was shown to transcriptionally regulate *CYP19A1,* a gene coding for aromatase, an enzyme that converts testosterone to estradiol. This intriguing relationship between RORA and sex hormones led us to propose a model in which RORA deficiency may lead to an elevation of testosterone and depletion of estradiol through suppression of *CYP19A1* expression. This model provides a plausible biochemical explanation for the elevated testosterone levels seen in studies by the Baron-Cohen group. It also suggests a more direct mechanism for the sex bias in ASD in which normal females, with higher estrogen levels, might exhibit higher expression of *RORA*, thus buffering against agents that induce RORA deficiency. This model further predicts that during development, *RORA* expression may be sexually dimorphic at least in some regions of the brain.

This pilot study was undertaken to examine the possibility of sex differences in the expression of *RORA* in several brain regions at different stages of development of the normal human brain using RNAseq data from the BrainSpan Atlas. In addition, sex differences in RORA and aromatase protein levels were investigated by reanalysis of our published confocal immunofluorescence data from the cortex of both male and female controls and age-matched male and female ASD donors. To reduce heterogeneity in the expression patterns due to the genetics as well as age of the brain donors, we also conducted expression analyses of the cortex and cerebellum of a strain of mice at 3 months of age. Together, these studies suggest sexual dimorphism in the expression of *RORA/Rora* in some brain regions during certain stages of development as well as a high correlation with the expression of its target genes, especially *CYP19A1*.

## Methods

### Re-analysis of pre-existing confocal immunofluorescence data from tissue arrays

Data from a previous study [30] employing confocal immunofluorescence to quantitate protein levels of RORA and aromatase on a tissue array containing specimens of the frontal cortex of ASD cases and age-matched controls were reanalyzed on the basis of gender. The tissue array was obtained through the Autism Tissue Program (San Diego, CA, USA) and kindly provided by Dr. Janine LaSalle (UC Davis). Each array contained 600 μm in diameter × 5 μm thick sections in triplicate from the BA9 region of the frontal cortex of autistic individuals and age- and sex-matched controls as well as samples from individuals with a variety of other neurodevelopmental disorders as previously described [31]. Only specimens from neurotypical controls (without any developmental disorder) were used as ‘Controls’ for this study. The amounts of protein are determined by calculating the ‘mean fluorescence’ for RORA and aromatase across 40 to 50 neurons per sample. Furthermore, comparisons of protein levels between females and males and between cases and controls are made for samples that are matched for donor age within ± 3 years. Additional file 1 contains the ages and mean fluorescence levels of antibody staining for RORA and aromatase in all of the ASD donors and controls from the tissue array.

### Analysis of RNAseq data from the BrainSpan atlas of the developing human brain

RNAseq data (RPKM) for gene expression of *RORA* and that of selected transcriptional targets of RORA in specific brain regions were downloaded from the BrainSpan Atlas of the Developing Human Brain [32]. The downloaded data for the orbital frontal cortex and the cerebellum are presented in Additional file 2. The samples, divided by gender, were grouped into three developmental periods: (1) before birth (BB), which was designated in BrainSpan as ‘pcw’ for post-conception weeks; (2) birth to 18 years of age; and (3) older than 19 years of age. Within each group, the samples were matched for age ± 3 years (or ± 3 pcw for the prenatal samples) for comparisons between males and females or between cases and controls. The validated transcriptional targets of RORA selected for RNAseq data analyses were *A2BP1*, *ITPR1*, and *NLGN1*, which are among the autism susceptibility genes represented in the SFARI gene and AutismKB databases [14, 15]. *CYP19A1* was not included in these analyses because the RNAseq values were either zero or too low for confidence. The functions of these genes in the context of ASD will be discussed later.

### Extraction of frontal cortex and cerebellum from wild-type C57BL/6 mice

Wild type C57BL/6 mice were obtained from our colony at the IFR 83 Biologie Integrative, UPMC (Paris, France). Animal housing and all handling procedures for this study followed ethical guidelines established by Le Comité National d’Ethique pour les Sciences de la Vie et de la Santé (animal ethics committee for France), in accordance with the European Communities Council Directive 2010/63/EU. Male and female mice (three per group) aged 3 months were euthanized with an overdose of sodium pentobarbital (300 mg/Kg i.p.) and the brain rapidly dissected into RNase-free normal saline (0.9% NaCl). Female mice were not synchronized with respect to estrous cycle before the brains were harvested. The frontal cortex and cerebellum were isolated and placed into 100 μL RNALater (Qiagen, France) and maintained at 4°C for 24 h prior to freezing at -80°C.

### RNA isolation and quantitative RT-PCR analysis

Mouse brain tissues were homogenized in a Bullet Blender Homogenizer (Next Advance, Averill Park, NY, USA), after which total RNA was isolated using an RNeasy Mini Kit (Qiagen, Gaithersburg, MD, USA). A total of 1 μg purified total RNA was used for cDNA synthesis using the iScript cDNA Synthesis Kit (BioRad, Hercules, CA, USA) according to the manufacturer’s protocols. The reaction (20 μL) was incubated at 25°C for 5 min, followed by 42°C for 30 min, and ending with 85°C for 5 min. After reverse transcription, the cDNA reaction mixture was diluted to a volume of 50 μL with nuclease-free water and used as a template for qPCR analyses. Real-time PCR analyses were conducted using the Applied Biosystems 7300 Real-Time PCR System (Applied Biosystems, Foster City, CA, USA). Each sample was run in triplicate and the average deviation of the CT values was calculated to assess the consistency of the assays. An average CT deviation of 0.25 or less was considered acceptable for replicates. Primers for RT-qPCR analyses were designed using Primer3 software for mouse *Rora* as well as *A2bp1*, *Cyp19a1*, *Itpr1*, and *Nlgn1*. The primer sequences for these mouse genes are listed in Additional file 3. *Cyp19a1* expression was not determined for the cerebellum because the expression levels were either zero or too low for confidence. Primers for the human genes have been previously published [33]. The relative quantity of transcripts in each sample was calculated using the standard curve method with *18S* RNA expression as a reference.

### Statistical analyses

The Student’s t-test within the StatPac Statistical Program (StatPac, Inc., Bloomington, MN, USA) was used to determine *t*-values and *P* values for differences between the means of gene expression in age-matched males and females and between the means of age-matched cases and controls. *T*-values derived from the t-tests and degrees of freedom (sum of the number of samples in two groups minus 2) were also used to determine Cohen’s *d* and effect size-*r* to provide an additional measure of the magnitude of the effect of sex (or autism diagnosis) on protein or gene expression level. Cohen’s *d* and effect size-*r* were calculated using the open-access online Effect Size Calculator http://www.uccs.edu/lbecker/index.html made available by Dr. Lee A. Becker (University of Colorado, Colorado Springs, CO, USA). StatPac was also used to determine correlation coefficients and *P* values for comparisons of gene expression levels for *RORA/Rora* and each of the transcriptional targets. Two-tailed *P* values are reported for all statistical analyses.

Power and sample size analyses for the experiments involving comparisons of independent group means (for example, case vs. control or female vs. male) were performed using the open-access online Power/Sample Size Calculator http://www.stat.ubc.ca/~rollin/stats/ssize/n2.html which is kindly provided by Dr. Rollin Brant (University of British Columbia, Vancouver, Canada). For power calculations involving a given number of samples in each group (n), a two-sided test was selected with α set to 0.05. The parameters used for sample size calculation were two-sided test, with α = 0.05 and power = 0.80.

All boxplots were generated using the open-source software BoxPlotR (http://boxplot.tyerslab.com) kindly made available online by the Tyers (IRIC-Universite de Montreal, Montreal, Quebec, Canada) and Rappsilber (University of Edinburgh, UK) Laboratories.

## Results

### Confocal immunofluorescence data on the human frontal cortex suggests lower *RORA*and aromatase protein levels in males

**Figure 1:**
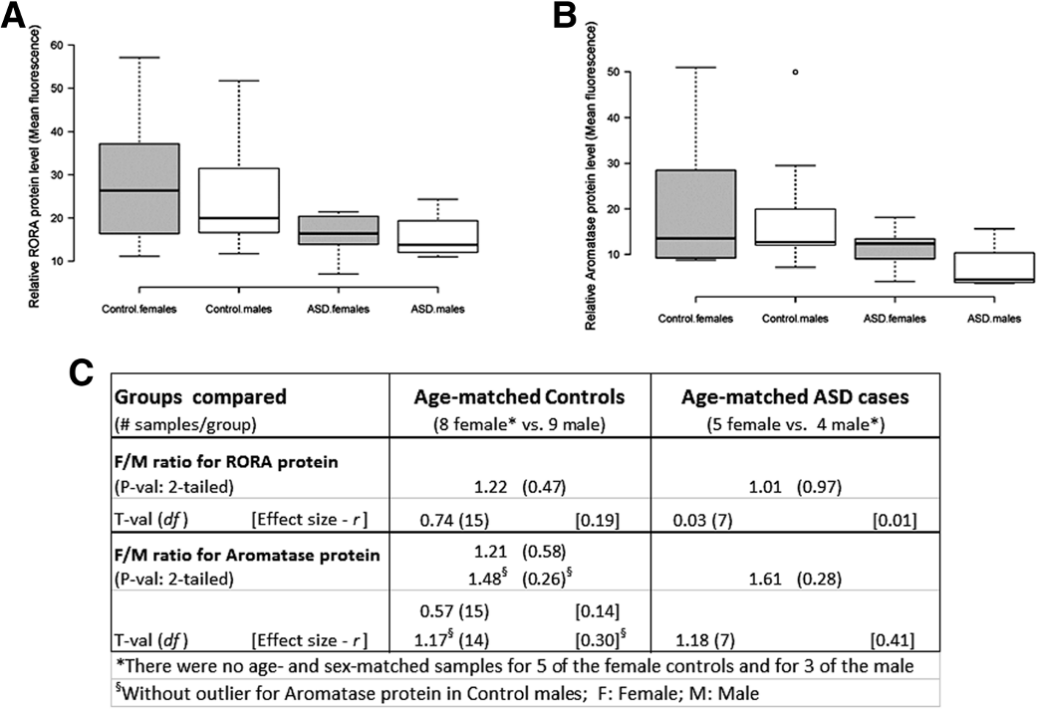
**RORA and aromatase protein in the postmortem frontal cortex (BA9) of females and males.** Comparison of RORA **(A)** and aromatase **(B)** protein levels in postmortem tissues from the frontal cortex of control females (n = 8) and age-matched control males (n = 9) as well as age-matched females (n = 5) and males (n = 4) with ASD. Results were obtained by re-analysis of data from confocal immunofluorescence analyses of tissue arrays previously reported by Sarachana *et al*. [30]. The dark bars in the boxes represent the medians, the box limits indicate the 25th and 75th percentiles as determined by the statistical software R, and the whiskers extend 1.5 times the interquartile range from the 25th and 75th percentiles. The open circle above the boxplot represents an outlier in the aromatase level for control males. The female-to-male (F/M) protein ratios (shown in **C**) were calculated based on the average mean fluorescence values for the respective groups, and two-tailed *P* values, t-values, and effect sizes are given for the comparisons between the protein levels in females and males.

**Figure 2:**
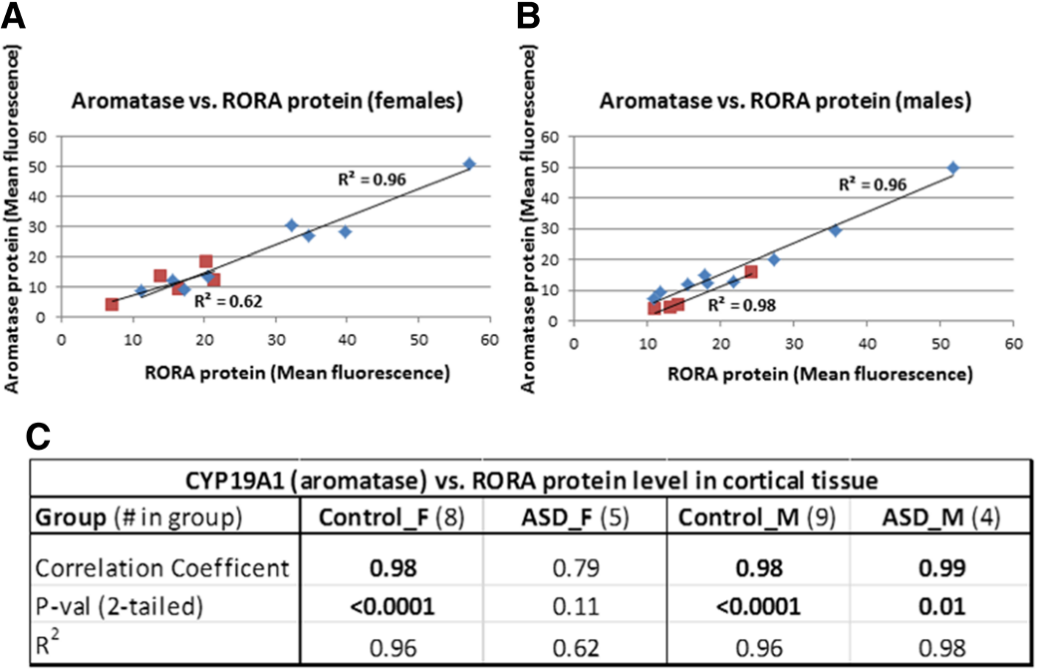
**Correlation between aromatase and RORA protein levels in the frontal cortex of age-matched male and female controls and age-matched male and female donors with ASD. (A)** Correlation between aromatase and RORA protein levels in control females (diamond shapes) and females with ASD (squares). **(B)** Correlation between aromatase and RORA protein levels in control males (diamond shapes) and males with ASD (squares). **(C)** The table shows the correlation coefficients, *P* values, and coefficient of determination (R^2^) values for the relationship between aromatase and RORA protein levels.

We have previously demonstrated reduced expression of RORA and aromatase protein in ASD cases relative to controls using confocal immunofluorescence analyses of tissue arrays containing postmortem frontal cortex specimens [30]. Here, we reanalyze the confocal immunofluorescence data from age-matched male (n = 9) and female (n = 8) controls and show that there is a nominally higher level (1.22-fold) of RORA protein in females relative to males (Figure 1). Similarly, the female-to-male (F/M) ratio for aromatase is 1.21. However, these modest differences are not statistically significant (two-tailed *P* >0.47 for RORA). There is no apparent sex difference in RORA protein in the cortex of male and female individuals with ASD (F/M = 1.01, *P* = 0.97, effect size = 0.01), while the F/M ratio for aromatase is 1.61 (*P* = 0.28). Table 1 summarizes the case-control comparisons of both RORA and aromatase protein levels for the combined (males + females) set of ASD and control specimens as well as for age-matched females and males. While the lower protein levels for the combined cases vs. combined controls are statistically significant as previously reported [30], the nominally lower levels of RORA and aromatase in tissues from cases are not statistically significant from that of controls when the samples are divided into age-matched females and age-matched males. Because the correlation between RORA and aromatase protein levels was previously found to be highly correlated for the combined samples (R^2^ = 0.91), we examined the correlation between these protein levels in both control samples and ASD samples as a function of sex. Figure 2 shows that the correlation between RORA and aromatase is very high for both control and ASD male samples (R^2^ ≥ 0.96; *P* <0.01) as well as for female controls (R^2^ = 0.96; *P* <0.0001), while R^2^ is only 0.62 (*P* = 0.11) for samples from ASD females.

**Table 1:** **Comparisons of Aromatase and RORA protein levels in the frontal cortex of cases (A) and controls (C) as a function of sex**

Case/control comparisons (no. of samples/group)	Combined (12 ASD; 22 controls)	Age-matched case-control females (5 ASD; 5 controls)	Age-matched case-control males (7 ASD; 6 controls)
**A/C Ratio of RORA protein**	0.68	0.77	0.78
(*P* val: 2-tailed)	(0.046)	(0.31)	(0.46)
T-val (*df*) [Effect size - *r*]	2.07 (32) [0.34]	1.09 (8) [0.36]	0.76 (11) [0.22]
**A/C Ratio of Aromatase protein**	0.56	0.72	0.55
(*P* val: 2-tailed)	(0.028)	(0.31)	(0.21)
T-val (*df*) [Effect size - *r*]	2.30 (32) [0.38]	1.11 (8) [0.36]	1.34 (11) [0.37]

A: ASD cases; C: Controls.

The table shows the case-control (A/C) ratio of RORA and Aromatase protein levels determined by confocal immunofluorescence analyses of tissue arrays containing specimens from the BA9 region of individuals with ASD and age-matched controls with no evidence of neurodevelopmental disorder. The *P* values, t-values, and effect sizes relate to the comparisons of protein levels for cases and controls, each divided by sex.

### Correlation of *RORA*and target gene expression in human postmortem brain tissues

Because of the strong correlation between RORA and aromatase protein levels on tissue arrays of cortical specimens from males and females, we examined the correlation between the mRNA expression of *RORA* and that of several validated transcriptional targets of RORA in postmortem tissues from the prefrontal cortex of male controls and ASD donors using RT-qPCR data from an earlier study which investigated genome-wide transcriptional targets of RORA [33]. Table 2 summarizes the results of the correlation analyses for the expression of *CYP19A1*, *A2BP1*, *ITPR1*, and *NGLN1* vs. *RORA* expression. As shown, the correlation coefficients are in the range of 0.72 to 0.99 for the control samples, indicating a relatively strong positive correlation between expression of each of these four genes and *RORA* expression. Interestingly, the correlation between *CYP19A1* and *RORA* expression is identically high and significant for both male controls and age-matched ASD males (not shown) as was seen for the protein correlation in these groups on tissue arrays (Figure 2), while the correlation between the other three target genes and *RORA* are much lower for cases (R^2^ ≤ 0.36).

**Table 2:** **Correlation coefficients for** ***RORA*** **and target gene expression in the postmortem frontal cortex of control males**

Comparison	CYP19A1 vs. RORA*	A2BP1 vs. RORA	ITPR1 vs. RORA	NLGN1 vs. RORA
Correlation coefficient	0.99	0.83	0.72	0.81
*P* val (2-tailed)	0.03	0.38	0.49	0.39
R^2^	0.99	0.69	0.52	0.66

The correlation coefficients between *RORA* expression and that of several of its target genes were derived from a re-analysis of previously published gene expression data [33]. The two-tailed *P* values for the correlations and the coefficient of determination (R^2^) are also shown. Ages of the control male donors were 19, 22, and 28 years. Not shown are the analogous correlation data for male donors with ASD, aged 20, 22, and 30 years.

*With the ASD group, the correlation coefficient, *P* value, and R^2^ was identical to that of the male controls for the relationship between *RORA* and *CYP19A1*, while the correlation between *RORA* expression and that of the other three target genes was low (R^2^ < 0.36).

### Expression of *RORA*and selected transcriptional targets of RORA across brain development based on RNAseq data from BrainSpan

As ASD is a developmental disorder, we examined gender-related developmental changes in *RORA* through meta-analyses of *RORA* expression using RNAseq data from the BrainSpan Atlas of Human Development [32]. Here, we analyzed the expression of *RORA* and its correlation with that of three of its transcriptional targets (*A2BP1*, *ITPR1*, and *NLGN1*) in two brain regions which are involved in ASD: the orbital prefrontal cortex and the cerebellum.

#### Orbital prefrontal cortex

**Figure 3:**
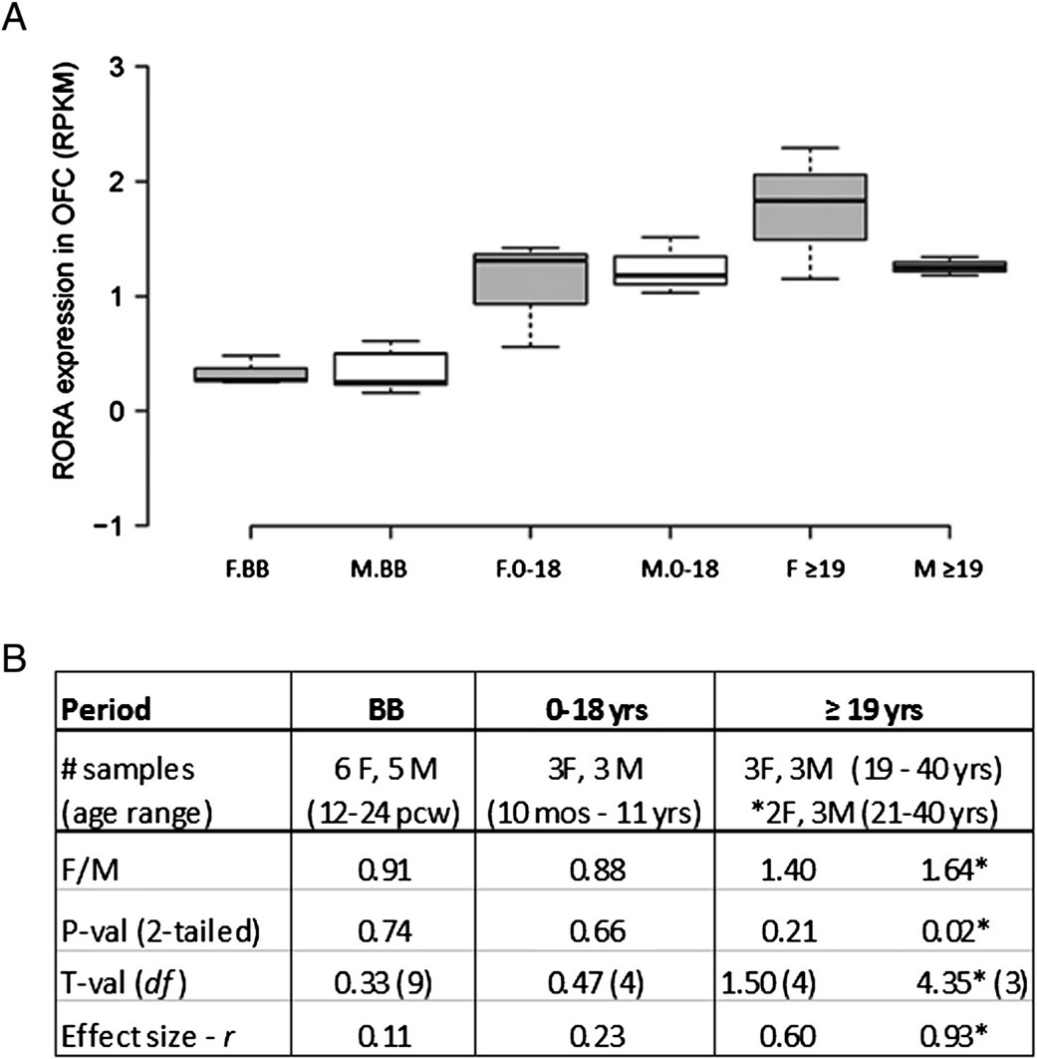
**Expression of** ***RORA*** **in the human orbital frontal cortex of age-matched females and males as a function of developmental stage. (A)** *RORA* expression in the orbital frontal cortex (OFC) of age-matched females (F) and males (M) before birth (BB), and at different intervals after birth (birth to 18 years and ≥19 years) using RNAseq data from the BrainSpan Atlas of the Developing Human Brain [32]. The number of females (F) and males (M) included in each developmental period were: BB (6 F:5 M), birth-18 (3 F:3 M), and ≥ 19 (3 F:3 M). **(B)** The table shows the female-to-male (F/M) expression ratios and associated two-tailed *P* values, t-values, and effect sizes for differences between the group means for different developmental periods. *For this comparison, a female whose age difference exceeded (by one year) the age-matching criteria of ± 3 years with respect to the male group was excluded from the analyses.

Although there is suggestive evidence for sexually dimorphic expression of *RORA* in the frontal cortex (presented above), at present, there is no known biological or anatomical correlate for this difference between females and males. However, because a recent magnetic resonance imaging study on brain morphometric differences between adult females and males reported increased volume in the orbital frontal cortex (OFC) of unaffected females in comparison to age-matched unaffected males [26], we examined *RORA* expression in this region across several developmental periods. Figure 3 shows that there are developmental differences but no significant sex differences in the average level of *RORA* expression in this brain region. There is, however, a suggestive difference in expression of *RORA* (F/M = 1.40; *P* = 0.21, effect size = 0.6) in the adult group (≥19 years of age), which is the age group represented in the imaging study by Lai *et al.*[26]. Table 3 and Additional file 4 show the correlation coefficients and R^2^ plots, respectively, for the expression of *RORA* and each of its three target genes in the OFC in the three defined developmental periods. For both females and males, there is very little correlation between *RORA* expression and that of the three target genes before birth, but relatively high correlation between *RORA* and all three genes in females in the 0 to 18 years age group. While the high correlation with *NLGN1* is maintained in the adult females, the correlation for two genes, *A2BP1* and *ITPR1*, decreases in this group. Males, on the other hand, have a much more variable pattern of correlations for *RORA* and these target genes in the OFC, in which each of the genes exhibits a high correlation with *RORA* expression, but in different postnatal periods and, in one case (*A2BP1*), in a negative direction.

**Table 3:** **Correlation of** ***RORA*** **and target gene expression in the orbital frontal cortex (OFC) of age-matched females and males**

**A2BP1 vs. RORA**	**Females**			**Males**		
Group (no. of samples)	F_BB (6)	F_0-18 (3)	F >19 (3)	M_BB (5)	M_0-18 (3)	M >19 (3)
Correlation coefficient	-0.68	0.99	0.35	-0.39	0.73	-0.99
*P* val (2-tailed)	0.13	0.08	0.77	0.51	0.48	0.09
R^2^	0.47	0.98	0.12	0.15	0.54	0.99
**ITPR1 vs. RORA**	**Females**			**Males**		
Group (no. of samples)	F_BB (6)	F_0-18 (3)	F >19 (3)	M_BB (5)	M_0-18 (3)	M >19 (3)
Correlation coefficient	0.36	0.99	0.72	0.29	0.96	-0.76
*P* val (2-tailed)	0.49	0.10	0.49	0.64	0.18	0.45
R^2^	0.14	0.97	0.52	0.08	0.92	0.54
**NLGN1 vs. RORA**	**Females**			**Males**		
Group (no. of samples)	F_BB (6)	F_0-18 (3)	F >19 (3)	M_BB (5)	M_0-18 (3)	M >19 (3)
Correlation coefficient	-0.26	0.97	1.00	−0.52	-0.03	0.95
*P* val (2-tailed)	0.61	0.16	0.02	0.37	0.98	0.21
R^2^	0.08	0.93	1.00	0.27	0.00	0.90

The correlation coefficients, *P* values, and R^2^ values are shown for *RORA* expression and that of *A2BP1*, *ITPR1*, and *NLGN1* across each of the three developmental periods.

#### The cerebellum

**Figure 4:**
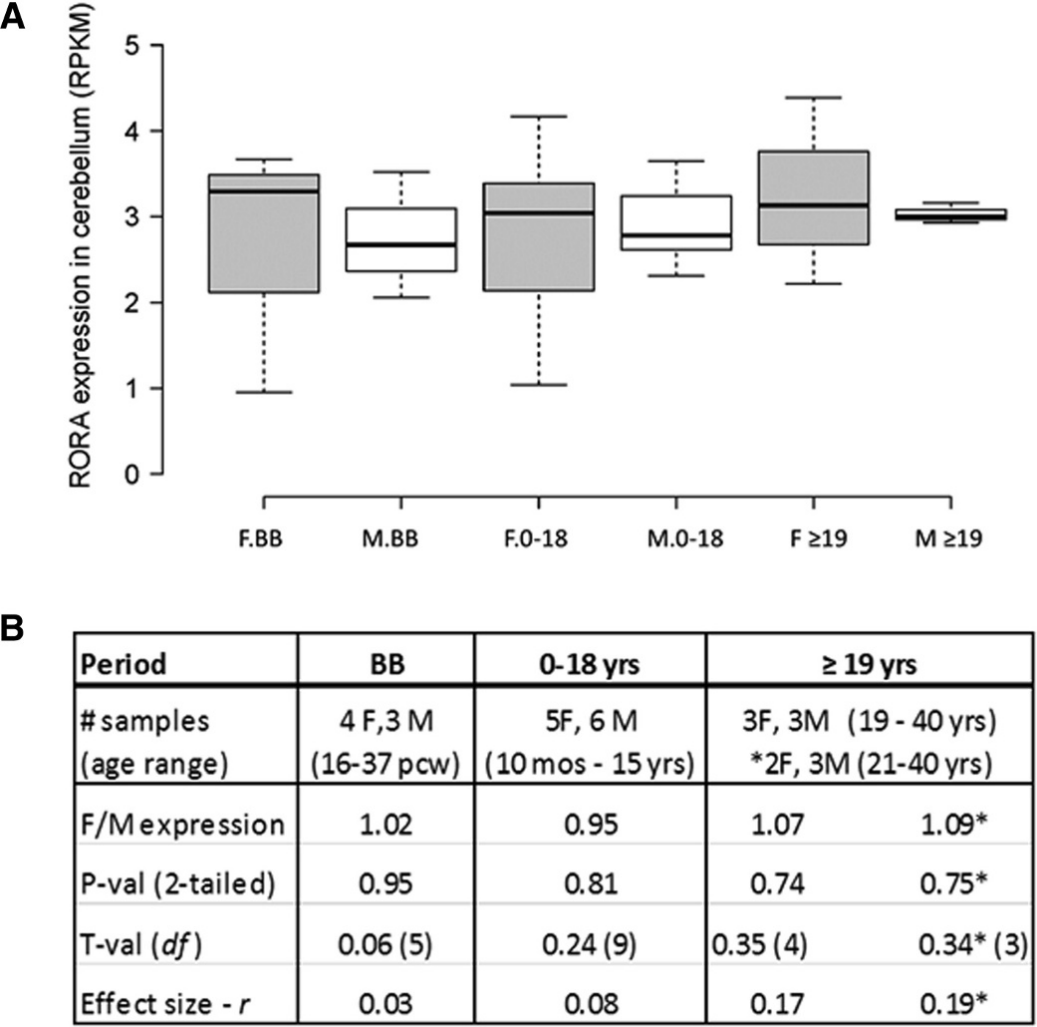
**Expression of** ***RORA*** **in the cerebellar cortex of age-matched females and males at different developmental stages. (A)** *RORA* expression was determined by a meta-analysis of RNAseq data from the BrainSpan Atlas of the Developing Human Brain. The total number of age-matched females (F) and males (M) included in each developmental period were: BB (4 F:3 M), birth to 18 years (5 F:6 M) and ≥19 years (3 F:3 M). **(B)** The table shows the female-to-male (F/M) expression ratios and associated two-tailed *P* values, t-values, and effect sizes for differences between the group means for different developmental periods. *For this comparison, a female whose age difference exceeded (by 1 year) the age-matching criteria of ± 3 years with respect to the male group was excluded from the analyses.

In order to further probe the sexually dimorphic expression of *RORA*, we also studied during several developmental periods the expression of *RORA* in the cerebellar cortex where it is known to play key roles in development and function [34–36]. Moreover, cerebellar pathology has been consistently reported in ASD [37]. Figure 4 shows that there were no significant sex differences in *RORA* expression in the cerebellum of age-matched males and females at any of these developmental periods. Table 4 and Additional file 5 further show the correlation data and graphs, respectively, for the expression of *RORA* and three of its target genes both before and after birth. Interestingly, the correlation between the prenatal expression levels of *RORA* and all three genes is significantly high (R^2^ ≥ 0.96, *P* ≤0.04) for females, but only significantly high for *RORA* and *A2BP1* in males. The postnatal female pattern of correlations between *RORA* and the three genes approximately mirrors the trend seen for correlations in the OFC in females, with high and significant correlations in the 0 to 18 years age group, and high correlations of lower significance in the adult group. The pattern of correlations in the male cerebellum after birth is more variable, as it was in the OFC.

**Table 4:** **Correlation of** ***RORA*** **and target gene expression in the cerebellum of age-matched females and males**

**A2BP1 vs. RORA**	**Females**			**Males**		
Group (no. of samples)	F_BB (4)	F_0-18 (5)	F >19 (3)	M_BB (3)	M_0-18 (6)	M >19 (3)
Correlation coefficient	0.96	0.95	0.82	1.00	0.64	-0.36
*P* val (2-tailed)	0.04	0.01	0.39	0.05	0.17	0.76
R^2^	0.93	0.91	0.67	0.99	0.41	0.14
**ITPR1 vs. RORA**	**Females**			**Males**		
Group (no. of samples)	F_BB (4)	F_0-18 (5)	F > 19 (3)	M_BB (3)	M_0-18 (6)	M > 19 (3)
Correlation coefficient	0.98	0.95	0.98	0.71	0.89	0.96
P-val (2-tailed)	0.02	0.02	0.14	0.49	0.02	0.17
R^2^	0.96	0.89	0.95	0.52	0.79	0.93
**NLGN1 vs. RORA**	**Females**			**Males**		
Group (no. of samples)	F_BB (4)	F_0-18 (5)	F > 19 (3)	M_BB (3)	M_0-18 (6)	M > 19 (3)
Correlation coefficient	0.99	0.99	0.99	0.90	0.42	0.41
P-val (2-tailed)	0.01	0.001	0.07	0.28	0.40	0.73
R^2^	0.97	0.98	0.99	0.83	0.18	0.17

The correlation coefficients, *P* values, and R^2^ values are shown for *RORA* expression and that of *A2BP1*, *ITPR1*, and *NLGN1* across each of the three developmental periods.

### Analysis of *Rora*and transcriptional target gene expression in the frontal cortex and cerebellum of wild-type male and female C57BL/6 mice

**Figure 5:**
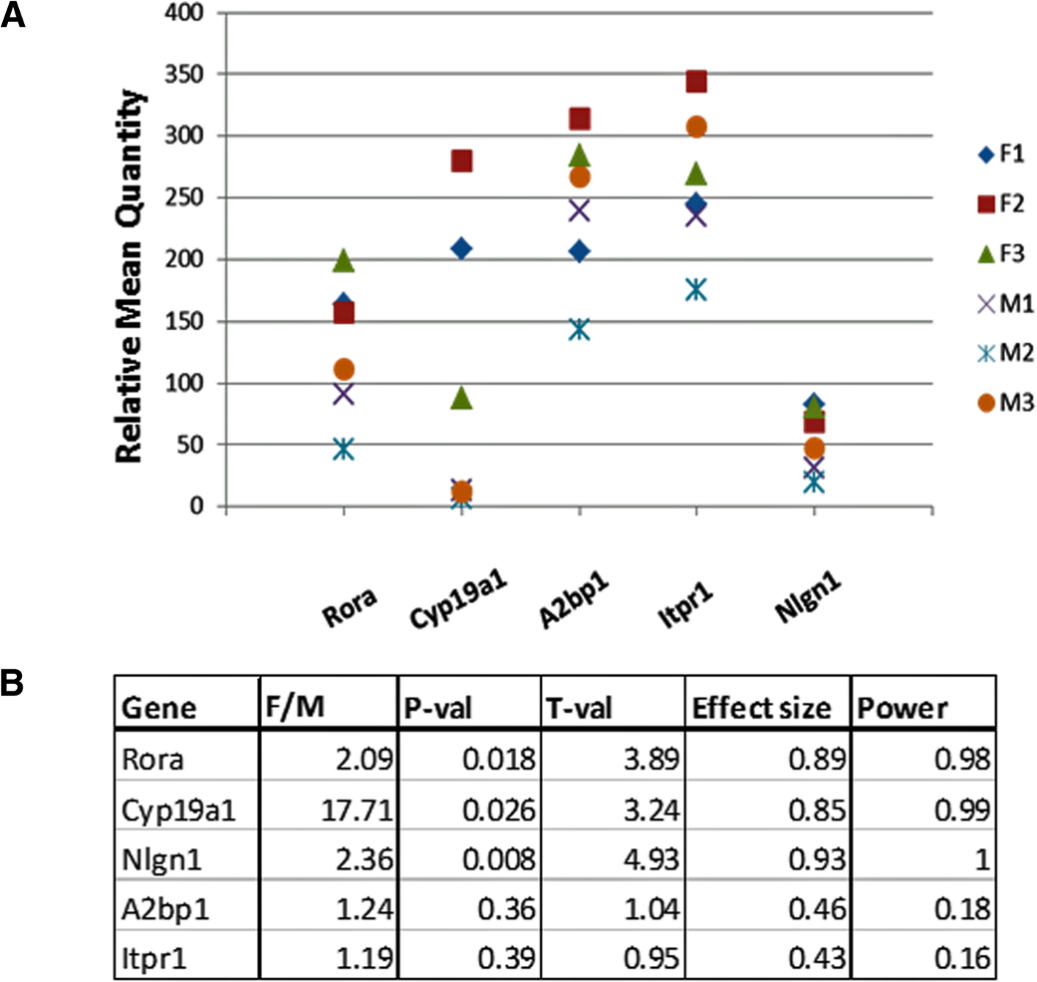
**Expression of** ***Rora*** and several of its transcriptional targets* in the frontal cortex of mice. (A) The scatter plot shows the relative transcript levels of *Rora* and several of its transcriptional targets (*Cyp19a1*, *A2bp1*, *Itpr1*, *Nlgn1*) in the frontal cortex of female (F) and male (M) C57BL/6 mice. There are three females and three males per group. **(B)** Table showing the female-to-male (F/M) expression ratios for each of the genes analyzed and two-tailed *P* values, t-values, effect sizes, and power for the comparisons of the group means for females and males. Using the means and standard deviations for the analyses, power was calculated for a two-sided *t*-test with three samples/group for which α was set at 0.05. *The transcriptional targets in mice were inferred based on *RORA* promoter binding analysis in SH-SY5Y cells [33].

Because the genetic heterogeneity of human subjects most likely influences gene expression [38], thus rendering direct comparisons of expression data from a limited number of postmortem samples (n = 3 to 6) insignificant or at best marginally significant, we conducted pilot studies with male and female wild-type C57BL/6 mice to address the question of sexually dimorphic expression of *Rora* and that of several of its validated transcriptional targets [33]. Figure 5 shows that, even with only three mice of each sex, there is a significant difference between the expression of *Rora*, *Cyp19a1*, and *Nlgn1* in the cortex of male and female mice (*P* = 0.008 - 0.026), with expression of all three genes being higher in the females. The strength of this association between gene expression and sex is also reflected in the large effect sizes (0.85 - 0.93) for these three genes. The expression of *A2bp1* and *Itpr1* is also nominally higher in females (F/M ratio approximately 1.2), but the *P* values for sex differences fall below the standard level of significance.

**Figure 6:**
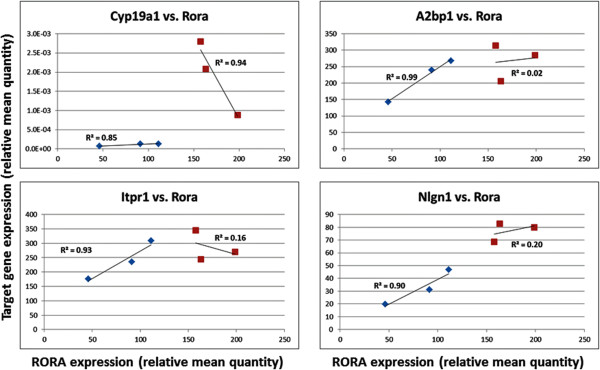
**Correlation between target gene expression and that of** ***Rora*** **in the frontal cortex of mice.** Red: females; Blue: males.

Table 5 and Figure 6 show the results of correlation analyses between the expression of *Rora* and that of three transcriptional targets in the mouse cortex. It is interesting to note that when separated by sex, the correlation of expression between *Rora* and each of the four targets represented here is much higher in males than in females, with correlation coefficients in the range of 0.92 to 0.99 (*P* values: 0.06 to 0.25), and R^2^ values (an indicator of effect size) in the range of 0.85 to 0.99. Also of interest is the strongly negative correlation coefficient (-0.97) between *Cyp19a1* and *Rora* in females.

**Table 5:** **Correlation of** ***Rora*** **and target gene expression levels in the frontal cortex of adult male and female mice**

Cyp19a1 vs. Rora	All	Males	Females
Correlation coefficient	0.63	0.92	-0.97
*P* val (2-tailed)	0.18	0.25	0.16
R^2^	0.4	0.85	0.94
**A2bp1 vs. Rora**	**All**	**Males**	**Females**
Correlation coefficient	0.68	0.99	0.13
*P* val (2-tailed)	0.13	0.06	0.92
R^2^	0.46	0.99	0.02
**Itpr1 vs. Rora**	**All**	**Males**	**Females**
Correlation coefficient	0.59	0.96	-0.40
*P* val (2-tailed)	0.22	0.18	0.74
R^2^	0.35	0.92	0.16
**Nlgn1 vs. Rora**	**All**	**Males**	**Females**
Correlation coefficient	0.96	0.95	0.45
*P* val (2-tailed)	0.002	0.2	0.7
R^2^	0.92	0.9	0.2

The correlation coefficients, *P* values, and R^2^ values are shown for *Rora* expression and that of *Cyp19a1*, *A2bp1*, *Itpr1*, and *Nlgn1*. Three male and three female mice were used for each group.

**Figure 7:**
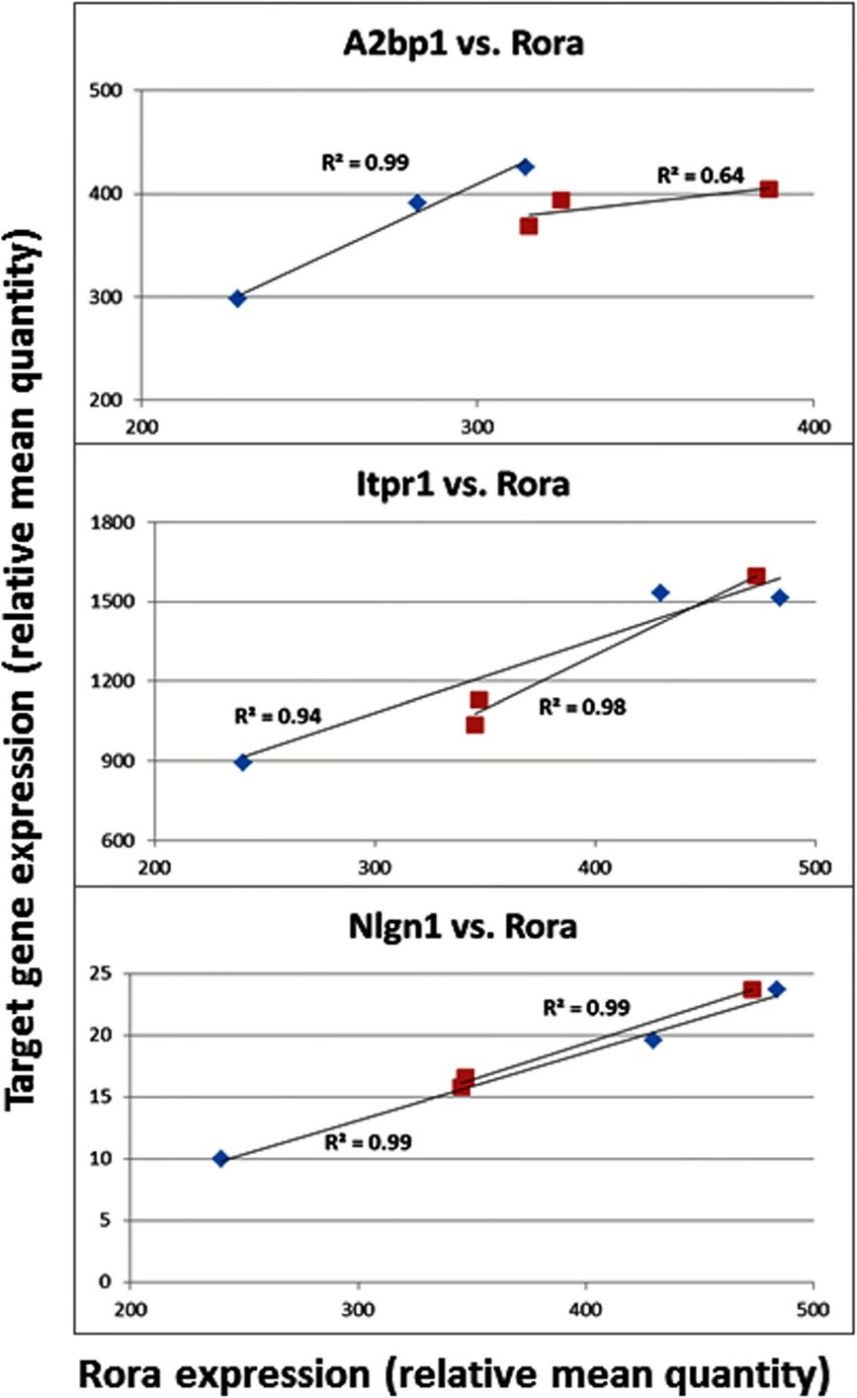
**Correlation between target gene expression and that of** ***Rora*** **in the cerebellum of mice.** Red: females; Blue: males.

With respect to the cerebellum, there were no significant sex differences in the expression of *Rora* and any of its target genes. This is comparable to the lack of significant sex differences in *RORA* expression in the human cerebellum. Table 6 and Figure 7 show that the correlation of *Rora* expression with that of the target genes is high for both males and females (R^2^ ≥ 0.94), with the exception of *A2bp1* in females, for which R^2^ is 0.64. It should be noted that the high correlation between the expression of *Rora* and its targets in both brain regions of adult male mice is distinctly different from the more variable patterns seen in the OFC and cerebellum of adult human males. On the other hand, the correlation of *Rora* expression with all three gene targets in the adult female mouse cerebellum is remarkably similar to that observed for the analogous correlations in the cerebellum of adult human females.

**Table 6:** **Correlation of** ***Rora*** **and target gene expression levels in the cerebellum of adult male and female mice**

A2bp1 vs. Rora	All	Males	Females
Correlation coefficient	0.74	1.00	0.80
*P* val (2-tailed)	0.09	0.07	0.41
R^2^	0.55	1.00	0.64
**Itpr1 vs. Rora**	**All**	**Males**	**Females**
Correlation coefficient	0.95	0.97	0.99
*P* val (2-tailed)	0.003	0.16	0.09
R^2^	0.91	0.94	0.98
**Nlgn1 vs. Rora**	**All**	**Males**	**Females**
Correlation coefficient	0.99	1.00	1.00
*P* val (2-tailed)	0.0001	0.05	0.04
R^2^	0.99	0.99	1.00

The correlation coefficients, *P* values, and R^2^ values are shown for *Rora* expression and that of *A2bp1*, *Itpr1*, and *Nlgn1*. Three male and three female mice were used for each group. The expression values for *Cyp19a1* in the cerebellum were either zero or too low to be confidently included in this analysis.

## Discussion

This exploratory study on sex differences in the ASD brain examines the sexually dimorphic expression of *RORA*, a functionally relevant candidate gene for autism, in the postmortem brain tissues of humans and mice, focusing on the frontal cortex and cerebellum. In addition, we investigated the correlation of *RORA* (or *Rora*) expression with several of its validated transcriptional targets in the same brain regions. For this study, we evaluated data for potential sex differences in *RORA/Rora* expression and/or its correlation with the expression of the transcriptional target genes from four sources: (1) our published confocal immunofluorescence analyses of tissue arrays containing cortical specimens from both male and female donors with ASD and age-matched controls [30]; (2) our prior gene expression analyses on frozen postmortem brain tissues from male controls and ASD donors [33]; (3) the collection of RNAseq data from the BrainSpan Atlas of the Developing Human Brain [32]; and (4) *de novo* expression analyses of *Rora* and the orthologous target genes in mouse brain tissues.

Although there was suggestive evidence for sexually dimorphic expression of *RORA* (F/M ratio approximately 1.2 - 1.4) in the human frontal cortex, these sex differences were not statistically significant. The lack of significance for sex differences in *RORA* expression may not be surprising given the genetic heterogeneity of human donors and the limited number of tissues available for analyses. In this regard, the effect sizes for sex and ASD influences on *RORA* expression and protein levels in the frontal cortex may be more informative because they are independent of sample size. Notably, the effect size for RORA protein level in male ASD cases vs. controls is lower (0.22) than the effect size for the female case–control comparison (0.36) (see Table 1). This difference suggests a smaller effect of ASD on RORA levels in males than in females, which may reflect a lower basal level of RORA in unaffected males relative to that in unaffected females. Interestingly, the effect size is virtually zero (0.01) for sex differences in RORA protein in ASD cases (see Figure 1), suggesting that both males and females affected by ASD have a comparably low level of RORA protein in the frontal cortex. In addition, the effect size for sex differences in *RORA* expression in the OFC of control males and females is at least 0.6 (see Figure 3). Thus, several determinations of effect size suggest that there may be a modest difference in the expression of *RORA* in the frontal cortex of control males and females which vanishes in males and females with ASD.

Perhaps of more relevance to the sex bias in ASD are sex differences in the correlation of *RORA* expression with that of some of its target genes in both the cortex and cerebellum. Studies with wild-type adult male and female C57BL/6 mice lend support to the existence of sex differences in *RORA* expression in the cortex and its correlation with target gene expression in these two brain regions. However, although the correlations between cortical expression of *Rora* and that of its transcriptional targets are higher in male mice in comparison to female mice, the analogous correlations using RNAseq data for human tissues are more complex and dependent on target gene and developmental period. It is also possible that the variability in correlations for human tissues is in part due to the genetic heterogeneity of the brain donors as well as the fact that the majority of age-matched samples used for the *RORA*-target expression correlations are not from the same donors. This donor disparity in the BrainSpan Atlas is in contrast to the samples involved in the confocal immunofluorescence and gene expression analyses performed by our laboratory, in which correlations of protein or gene expression levels (Figure 2 and Table 2, respectively) were determined for RORA and targets in the same samples, resulting in consistently higher and positive correlations. The following sections first highlight the principal findings of this study, and then discuss the implications of these findings with respect to the potential contribution of *RORA* to the sex bias and pathobiology of ASD.

### Comparison of RORA and aromatase protein levels in the frontal cortex

Although there were no statistically significant sex differences in RORA and aromatase protein levels in the cortical specimens on the tissue array (Figure 1), the correlation analyses show that the protein levels for both RORA and aromatase in ASD cases (age-matched females and males) fall at the lower end of the range of protein levels for control females and males (Figure 2). However, the correlation between these protein levels is higher for ASD males than for ASD females, which may relate to sex differences in the response to RORA deficiency. Notably, there is a greater percent reduction in aromatase protein in ASD males in comparison to ASD females, suggesting that females may be better able to offset aromatase deficiency caused by RORA deficiency through compensatory mechanisms.

### Correlation of gene expression levels in the frontal cortex

Table 2 reveals a significantly high correlation between the expression of *RORA* and that of *CYP19A1* (the gene coding for aromatase) in frozen postmortem cortical tissues from both control and ASD males. This high correlation at the level of gene expression thus reflects the high correlation seen at the protein level for male cases and controls. The strong and positive correlation between CYP19A1 and RORA protein levels suggests that a deficiency in aromatase is likely to result in higher testosterone (and lower estradiol) levels in the presence of RORA deficiency. Physiological disturbances in either of these hormones during critical periods of development may result in impaired neurological development and function, as discussed later.

### Analysis of RNAseq data for RORA and several of its transcriptional targets in the orbital frontal cortex (OFC) and cerebellum

RNAseq data from the BrainSpan Atlas was used as an experimental tool to explore sexually dimorphic expression of *RORA* and that of its target genes in different human brain regions across different developmental periods. Although there were no statistically significant sex differences in *RORA* expression in the OFC and cerebellum (Figures 3 and 4), there was a suggestively higher expression (and a correspondingly larger effect size) in the OFC of adult females in comparison to age-matched males, with an F/M ratio comparable to that observed for RORA protein in the frontal cortex (Figure 1). Of potentially greater interest are the sex differences in the correlation between *RORA* expression and that of its transcriptional targets, specifically *A2BP1 (RBFOX1)*, *ITPR1*, and *NLGN1*, summarized in Tables 3 and 4. However, the sex-dependent correlations are complex, being dependent on both brain region and period of development. In the OFC before birth where the expression of *RORA* is very low (approximately 10% of that in the cerebellum), there is virtually no correlation between *RORA* expression and that of any of the three target genes in either females or males. This suggests that RORA may not play a major role in the prenatal development of this brain region, in contrast to the critical role of RORA in development of the cerebellum [34], where the level of *RORA* expression is consistently high in both males and females across all developmental stages. The correlation patterns in the cerebellum are also different from the OFC, with females exhibiting significantly high correlations between *RORA* expression and that of the target genes through age 18, while the correlation pattern is more variable for males (Table 4), with each target gene showing a high correlation in at least one developmental period. Because some of this variability may be due to the mismatch of tissue donors (though age-matched) for the RNAseq studies which may confound correlation analyses, we performed gene expression analyses of *Rora* and the orthologous target genes in the cortex and cerebellum of a strain of adult male and female mice.

### Sexually dimorphic expression of Rora and its transcriptional targets in the mouse brain

The strongest evidence for sexually dimorphic expression of *RORA* in the brain is provided by the pilot studies using wild-type adult male and female C57BL/6 mice. Here, we show sex-dependent differences in the cortical expression of *Rora* and two of its transcriptional targets, *Cyp19a1* and *Nlgn1*, with expression of all three being significantly higher in females, which is also consistent with the large effect sizes for these genes. Power analyses, based on the expression data for these three genes, indicate sufficient power (≥0.98) to detect significant differences, even with only three mice per group. In part, these results using genetically homogeneous female and male mice support the sex differences in RORA protein that were suggested by our confocal immunofluorescence analyses of postmortem tissue specimens from the frontal cortex of males and females as well as that suggested by RNAseq data from the OFC of donors ≥19 years of age. However, there were no significant differences in *Rora* and target gene expression in the mouse cerebellum, mirroring the results of our analyses of the RNAseq data from the human cerebellum.

### Correlation of Rora and target gene expression in the mouse brain

Unlike the variable correlations of *RORA* and target gene expression in the brain of genetically heterogeneous human males and females, the correlations derived from the adult male and female mouse brain may be more informative and relevant to sex differences that potentially impact brain function. Interestingly, the correlation coefficients are high between *Rora* and all targets (including *Cyp19a1*) in the male cortex but variable and generally lower in the female cortex (Table 5). In contrast, the *Rora*-target gene correlations in the cerebellum are comparably high for both males and females with respect to all targets except for *A2bp1* in females (Table 6). Based on the more than 2,500 putative transcriptional targets of RORA that were identified by our previous chromatin immunoprecipitation-promoter chip hybridization (ChIP-on-chip) analyses [33], the higher correlation between the expression levels of *Rora* and each of its transcriptional targets suggests that Rora deficiency may have a greater impact on neurological development and function in males than in females, especially in the cortex. This is particularly relevant since over 400 of the identified transcriptional targets of RORA are listed in SFARI Gene and AutismKB databases as autism candidate/susceptibility genes [14, 15]. Moreover, RORA and estrogen receptor (ER) are both nuclear receptors that share the same DNA consensus binding sites and regulate the transcription of some of the same genes [39]. Thus, the higher concentration of estrogen, which has been demonstrated even in prepubescent females versus age-matched males [40], may offset the loss of transcriptional control due to RORA deficiency by activating ER-mediated transcription, resulting in a lesser impact on neurological development in females. Also intriguing is the strong negative correlation coefficient between *Rora* and *Cyp19a1* expression levels in female mice. In contrast to the expected increase in *Cyp19a1* (aromatase) expression with increasing *Rora* expression which is seen in males, it is possible that aromatase expression *in vivo* is instead tightly regulated to maintain estrogen homeostasis (that is, to prevent overproduction of estrogen) in females under normal conditions. Such homeostatic regulation of estrogen in females is particularly important inasmuch as RORA and ER share overlapping transcriptional targets.

#### Complex regulation of *RORA*expression in the brain

Regardless of sex differences, the underlying mechanisms driving regional and developmental differences in *RORA* expression, such as those seen in the OFC, are unclear. Recently, we described differential recruitment of co-regulator proteins that are involved in the sex hormone-dependent regulation of *RORA* in the SH-SY5Y neuronal cell model [41]. We demonstrated that estrogen-mediated upregulation of *RORA* expression required both the estrogen receptor (ER-alpha) and the coactivator NCOA5, while androgen (DHT)-mediated downregulation of *RORA* required the androgen receptor (AR) and the corepressor function of SUMO1. Thus, the regional and temporal patterns of *RORA* expression in males and females during development may reflect both the tissue-dependent and developmental patterns of co-regulator and/or sex hormone receptor expression in the respective brain regions. Likewise, transcriptional regulation of target genes by RORA, a nuclear receptor whose function requires interaction with co-regulator proteins, is also dependent on the regional and temporal patterns of co-regulator expression.

#### A model connecting *RORA*to the sex bias in autism

Based on our previous studies showing the reciprocal regulatory relationship between RORA and the sex hormones, we proposed a mechanistic model in which RORA deficiency may biochemically explain the increase in testosterone in ASD, which has been reported by Baron-Cohen and colleagues [21, 23]. The strong correlation between RORA and aromatase protein and gene expression levels in the human brain from our earlier study [30] and this study lends support to this model explaining, in part, the higher testosterone levels associated with some cases of ASD. However, the question regarding a direct role for RORA in the sex bias of ASD remains unresolved since there were no significant sex differences in *RORA* expression, despite a marginally higher level of *RORA* expression in the frontal cortex of normal adult females. Indeed, whether a 20% to 40% higher level of *RORA* expression in females can protect against genetically, physiologically, or environmentally-induced RORA deficiency during critical periods of development remains to be determined, possibly by using gene-knockdown animal models. It is noted that the levels of RORA protein in the cortex of male and female individuals with ASD are quite similar (Figure 1), perhaps reinforcing the idea of a ‘higher threshold for ASD in females’ which may include greater tolerance to RORA deficiency down to a level at which some of the pathobiological processes associated with ASD are triggered. At present, it is unclear what the functional consequences might be for sex differences in *RORA* expression, particularly in the adult OFC. However, given the known neuroprotective function of RORA [42–46], it is worth noting that the reported volume of the OFC is greater in female than in male adults [26]. With respect to sex bias in ASD, perhaps of more importance than *RORA* expression *per se* is the correlation between *RORA* expression and that of its transcriptional targets, the functions of which are described in the next section.

#### RORA, a molecular link between sex hormones, neurodevelopment, and autism pathobiology

The involvement of sex hormones in brain development has been well-documented [47–50], yet the precise molecular mechanisms through which the hormones affect neuronal processes, such as neurogenesis, neuron migration, synaptogenesis, synaptic plasticity, axon guidance, and dendrite formation, are not well understood. Equally unclear are the mechanisms through which the sex hormones (in particular estradiol) mediate the observed sexually dimorphic effects on dendritic spine formation and synaptogenesis in certain brain regions during development [51, 52]. A reasonable assumption is that these hormones act through their respective hormone receptors. However, although there is little evidence for genetic or functional alterations of the primary estrogen and androgen receptors (ERα and AR) in a significant number of ASD cases, homeostatic imbalance of their circulating hormones (for example, through altered aromatase expression) will inevitably alter receptor activation.

We suggest that RORA may be a molecular link between the sex hormones and neurodevelopment as well as a mediator of at least some of the pathobiological processes associated with autism. While RORA is a known regulator of circadian rhythm [53] which in turn has been linked to synaptic regulation [54], it is also a nuclear hormone receptor that is involved in the transcriptional regulation of many genes in different tissues, thus exhibiting tissue-dependent pleiotropic effects. In a neuronal cell model, we have demonstrated that RORA can bind to the promoter regions of over 2,500 genes, 438 of which are included in autism gene databases [14, 15]. Gene ontology analyses of the putative gene targets of RORA revealed significant overrepresentation of genes involved in neuronal differentiation, neuron projection morphogenesis, axonogenesis, and axon guidance. We validated six transcriptional targets of RORA (*A2BP1*, *CYP19A1*, *HSD17B10*, *ITPR1*, *NLGN1*, and *NTRK2*) and, in this study, investigated the correlation between the expression of *RORA* and that of four of these targets in both the human and mouse brain. With regard to functional relevance, *A2BP1*, also known as *RBFOX1*, codes for a neuron-specific splicing factor associated with synaptic transmission, neurodevelopment, and developmental delay [55, 56]. Furthermore, *A2BP1/RBFOX1* has been recently highlighted by a whole exome sequencing study as an evolutionarily constrained gene that regulates the splicing of a large number of other genes identified with high-confidence as ASD risk genes [57]. Interestingly, this study, which utilized a novel statistical model that integrated transmission and *de novo* association (TADA) analyses, also identified NLGN1 as one of the synaptic proteins encoded by the TADA genes, which included a number of other still unvalidated transcriptional targets of RORA (specifically, *ANK2*, *APH1A*, *CACNA1D*, *HOMER*, *MYO9B*, *NR3C2*, and *TRIO*). These strong genetic associations of putative RORA targets with ASD risk further suggest that RORA deficiency may have a large impact on neuronal functions disrupted in autism. Other neurologically relevant validated targets of RORA include ITPR1, a calcium signaling molecule involved in synaptogenesis, plasticity, dendritic contact, and long-term depression [58, 59], and NTRK2, a neurotrophin kinase also involved in axon guidance, synaptogenesis, plasticity, mood disorder, and learning [60, 61]. On the other hand, *CYP19A1* and *HSD17B10* both code for metabolic enzymes involved in the conversion of testosterone to estradiol, suggesting that RORA deficiency may inhibit both biochemical pathways, thus exacerbating the expected increase in testosterone or depletion of estradiol. At present, it is not known what homeostatic mechanisms might restore the hormonal balance when both of these pathways are inhibited. Moreover, although our model predicts that testosterone levels would increase when RORA (and aromatase) are decreased, there is no information to date on the sex hormone levels in brain tissues that are RORA-deficient. It should be mentioned that, although the sex hormone status and possible hormonal imbalance in the heterozygous staggerer (Rora^+/sg^) mouse has been discussed at length by Doulazmi *et al*. [45], our study on the regulation of *RORA* by sex hormones as well as the regulation of *CYP19A*1 by RORA only recently demonstrated these associations in a neuronal cell model [30]. With respect to neurological functions, *CYP19A1* (aromatase) is associated with neurogenesis, neuronal differentiation, synaptic plasticity, and social cognition [62–65], while *HSD17B10* is associated with mitochondrial integrity, mental retardation, and language impairment [66–70], therefore extending the impact of their dysregulation beyond endocrine metabolism. Thus, the neuronal activities and high level neurological functions associated with these validated transcriptional targets of RORA suggest a mechanism for induction of autism brain pathology driven by sex hormones under conditions of RORA deficiency. Finally, we suggest that RORA deficiency may be the direct result of genetic [71] and epigenetic modifications of *RORA*[29], and/or gene-environment interactions. With respect to GxE interactions, *RORA* expression may be dysregulated by intrinsic metabolic or physiological conditions (for example, sex hormone imbalance due to alterations in the steroid hormone biosynthetic pathways [20, 28, 72]) or by extrinsic environmental factors, such as endocrine disrupting compounds, which are known to interfere with normal hormonal signaling [73–76].

#### Limitations and future directions

While this exploratory study provides suggestive evidence for sexually dimorphic expression of *RORA* in certain brain regions during development, the major limitation is a lack of sufficient postmortem brain samples to reveal statistically significant expression differences between males and females. Power analyses, based on the human data reported in this study, indicate that at least 121 samples per group would be necessary to significantly detect a 20% difference in the level of *RORA* expression between males and females with a power of 0.8. The lack of sufficient samples is further compounded by the need to divide the available samples into subgroups to evaluate sex-dependent differences in gene expression across development.

Another confounding factor is the genetic and phenotypic heterogeneity of the brain donors as there is ample evidence that genetics influences gene expression which, in turn, controls phenotype. Indeed, aside from not having ASD or any other diagnosed neurodevelopmental disorder, the behavioral phenotypes of the control brain donors are unknown. This pilot study using BrainSpan data will help to direct attention to certain brain regions and developmental stages in which sex differences in the expression of *RORA* and/or regulation of its target genes are suggested. This study also suggests that any study of sex-dependent differences in gene expression in the brain must take into account both regional and developmental changes in gene expression.

Regarding the issue of genetic heterogeneity, mouse strains are much more homogeneous with respect to genotype, but individual expression differences are still apparent even within a strain. Some of these differences, especially in post-pubertal females, may be due to hormonal cycling, so future studies on sex differences in gene expression should utilize more hormonally-synchronized female mice. At present, it is not possible to predict how estrous synchronization of female mice would affect sex hormone levels in the brain and the impact on neuronal expression of *Rora*. It is further noted that the effects of sex steroids in the brain are also mediated by the local synthesis of neurosteroids [63], and may not be directly correlated to circulating hormonal levels. Interestingly, there is greater variance in *RORA* expression among the brain tissue samples from human females included in this study, especially in the later stages of development. In analogy to studies with human brain tissues, studies using animal models to study sex differences in gene expression should also include different brain regions and developmental periods.

Finally, despite the obvious advantages of studying sex-dependent differences in gene expression in an animal model where genotype and other physiological conditions can be better controlled to reduce heterogeneity, it should be kept in mind that regulation of gene expression in the human brain is likely to be much more complex than in mouse brain. This complexity is in part suggested by the differences in correlation coefficients for the expression of *RORA/Rora* and its respective target genes in male humans and male mice, where the correlation coefficients are generally higher in the mouse. Some of these differences may be due to species-dependent differential expression and recruitment of co-regulators or hormone receptors, and/or species differences in epigenetic regulation and alternative splicing.

## Conclusions

Through analyses of pre-existing confocal immunofluorescence and gene expression data from our laboratory and publicly available RNAseq data, we present suggestive evidence that *RORA* may exhibit sex-dependent differences in gene expression in the human brain that are dependent on both brain region as well as stage of development. Significant sex differences in *Rora* and target gene expression are more readily observed in the cortex of a genetically homogeneous mouse model. With respect to gene expression in the mouse cortex, the stronger correlation between *Rora* and target gene expression in male mice in comparison to female mice suggests that Rora deficiency may have a greater impact on downstream events affecting neurological development and function in males in comparison to females. While sex differences in the correlation of *RORA* expression with that of its gene targets in the human brain are more complex, the consistently strong correlation between RORA and CYP19A1 protein and gene expression levels in our small number of postmortem human samples supports our proposed model for the involvement of RORA deficiency in the higher testosterone levels associated with increased risk for ASD.

## Electronic supplementary material

Additional file 1: **Samples and data from confocal immunofluorescence analyses.** Demographic and mean fluorescence data for tissue samples analyzed by confocal immunofluorescence. (PDF 59 KB)

Additional file 2: **RNAseq data from BrainSpan.** RNAseq data from the BrainSpan Atlas of the Developing Human Brain which was used for the meta-analyses of gene expression of RORA and transcriptional targets in the OFC and cerebellum. (XLSX 18 KB)

Additional file 3: **Primers for mouse expression analyses.** Primer sequences for qPCR analyses of *Rora* and its transcriptional targets in mouse tissues. (PDF 56 KB)

Additional file 4: **Correlation plots for** ***RORA*** **-target gene expression in the OFC.** (PDF 539 KB)

Additional file 5: **Correlation plots for RORA-target gene expression in the cerebellum.** (PDF 559 KB)

## Abbreviations

A2BP1(A2bp1)*: Ataxin 2 binding protein 1 (Also known as RBFOX1) *(mouse homologue of genes in parenthesis)
ASD: Autism spectrum disorder
BA: Brodmann area
ChIP: Chromatin immunoprecipitation
ChIP-on-chip: Chromatin immunoprecipitation followed by microarray analysis
CYP19A1(Cyp19a1): Cytochrome P450, family 19, subfamily A, polypeptide 1
HSD17B10: Hydroxysteroid (17-beta) dehydrogenase 10
ITPR1(Itpr1): Inositol 1,4,5-trisphosphate receptor, type 1
LCL: Lymphoblastoid cell line
NLGN1(Nlgn1): Neuroligin 1
NTRK2: Neurotrophic tyrosine kinase, receptor, type 2
qPCR: Quantitative polymerase chain reaction
RORA(Rora): Retinoic acid receptor-related orphan receptor alpha
RT: Reverse transcription
SH-SY5Y: Human neuroblastoma cell line.
