# Supplementary material for: Investigation of sex differences in the expression of RORA and its transcriptional targets in the brain as a potential contributor to the sex bias in autism
Source: Mol Autism. 2015 May 13;6:7. doi: 10.1186/2040-2392-6-7 (PMC4459681; doi:10.1186/2040-2392-6-7)
Supplement: Supplementary file 1 — Additional file 1: Samples and data from confocal immunofluorescence analyses. Demographic and mean fluorescence data for tissue samples analyzed by confocal immunofluorescence. (PDF 59 KB) [file 13229_2014_162_MOESM1_ESM.pdf]

**Males (A:ASD and C:Controls)**				
Diag	ID	Age	RORA	Aromatase
A	4849	7	10.99	3.72
A	4721	8	14.36	5.00
A	4899	14	13.12	4.09
A	B5144	20	24.35	15.62
A	B5000	27	15.99	13.45
A	B5173	30	24.09	11.55
A	B4498	56	28.04	25.47
C	229	4	51.70	49.97
C	1065	15	11.72	9.54
C	1027	22	17.77	15.14
C	602	27	15.45	12.03
C	1029	29	10.80	7.19
C	1104	35	27.32	19.93
C	1135	42	21.71	12.68
C	B4192	46	18.28	12.36
C	B4503	56	35.63	29.52

**Females (A:ASD and C:Controls)**				
Diag	ID	Age	RORA	Aromatase
A	4671	4	16.40	9.03
A	1182	9	21.41	12.36
A	B5342	11	20.40	18.14
A	3924	16	7.11	4.11
A	1638	20	13.91	13.44
C	1275	2	37.34	28.55
C	1377	5	34.64	27.11
C	3858	9	16.18	11.02
C	662	12	15.50	12.42
C	812	18	19.50	19.82
C	1486	22	17.28	9.21
C	1171	27	11.14	8.76
C	1136	33	32.20	30.26
C	1406	38	20.70	12.41
C	4643	42	20.58	13.51
C	4640	47	57.09	50.99
C	1941	52	34.43	23.29
C	4238	55	39.73	28.46
