# Supplementary material for: Investigation of sex differences in the expression of RORA and its transcriptional targets in the brain as a potential contributor to the sex bias in autism
Source: Mol Autism. 2015 May 13;6:7. doi: 10.1186/2040-2392-6-7 (PMC4459681; doi:10.1186/2040-2392-6-7)
Supplement: Supplementary file 3 — Additional file 3: Primers for mouse expression analyses. Primer sequences for qPCR analyses of Rora and its transcriptional targets in mouse tissues. (PDF 56 KB) [file 13229_2014_162_MOESM3_ESM.pdf]

Additional File 3: qPCR primers for mouse *Rora* and four transcriptional target genes in mice

Sequence Name	Sequence (5'-->3')
18s_F	GTT GGT GGA GCG ATT TGT CT
18s_R	AAC GCC ACT TGT CCC TCT AA
A2bp1_F	GAC CCC TAC CAC CAC ACA CT
A2bp1_R	GAA AGA ACG AGA CCC ACA TCA
Cyp19a1_5'_F	TGT TCT TGG AAA TGC TGA ACC
Cyp19a1_5'_R	AAT CTG CCA TGG GAA ATG AG
Itpr1_F	GGA ACA GAA CGA GCT GAG GA
Itpr1_R	AGG CCG ATT CTT TGT TTC TG
Nlgn1_F	GGG ATG AGG TTC CCT ATG TGT
Nlgn1_R	GGT TGG GTT TGG TAT GGA TG
Rora_F	GAA CCA CCG AGA AGA TGG AA
Rora_R	AGG AAA ATG GAG TCG CAC AA

F: Forward primer
R: Reverse primer
