# Supplementary material for: Investigation of sex differences in the expression of RORA and its transcriptional targets in the brain as a potential contributor to the sex bias in autism
Source: Mol Autism. 2015 May 13;6:7. doi: 10.1186/2040-2392-6-7 (PMC4459681; doi:10.1186/2040-2392-6-7)

Additional file 4. Correlation of RORA and target gene expression in the orbital frontal cortex (OFC) at different stages of development.

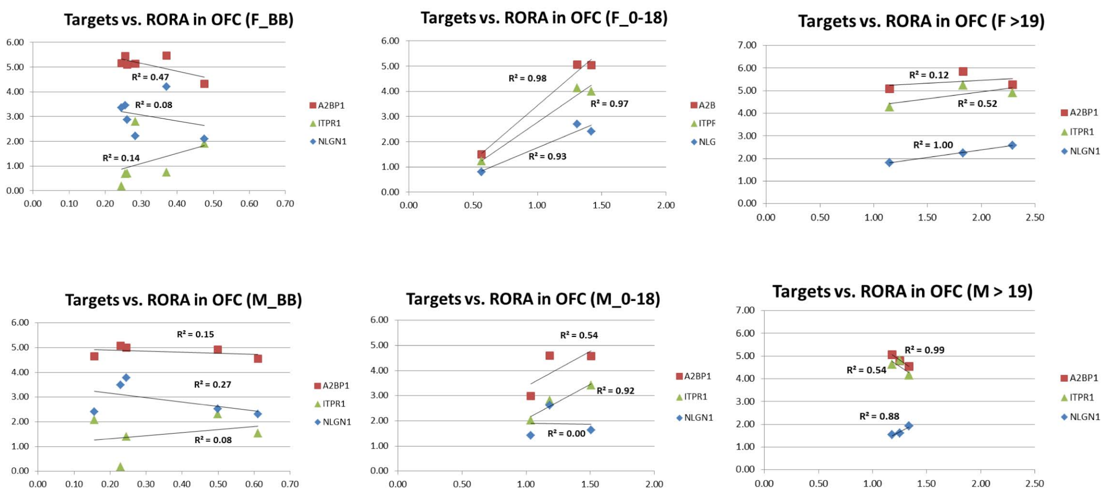

Supplement: Supplementary file 4 — Additional file 4: Correlation plots for RORA -target gene expression in the OFC. (PDF 539 KB) [file 13229_2014_162_MOESM4_ESM.pdf]
