# Supplementary figures and images for: Investigation of sex differences in the expression of RORA and its transcriptional targets in the brain as a potential contributor to the sex bias in autism
Source: Mol Autism. 2015 May 13;6:7. doi: 10.1186/2040-2392-6-7 (PMC4459681; doi:10.1186/2040-2392-6-7)

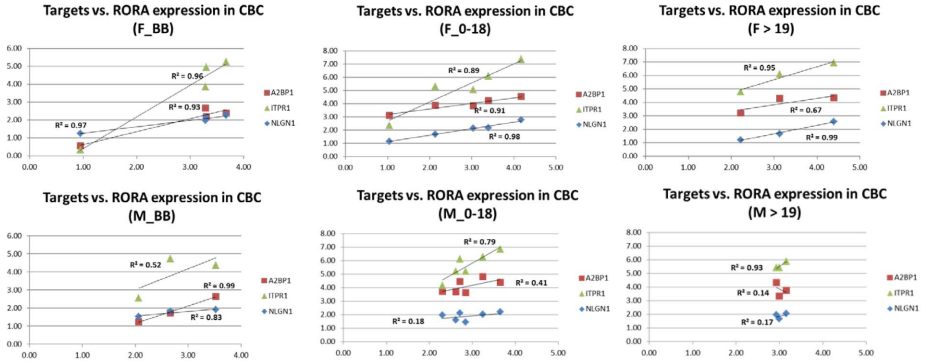

Supplement: Supplementary file 5 — Additional file 5: Correlation plots for RORA-target gene expression in the cerebellum. (PDF 559 KB) [file 13229_2014_162_MOESM5_ESM.pdf]
